# Multilayer Electrospun Scaffolds of Opposite-Charged Chitosans

**DOI:** 10.3390/ijms25063256

**Published:** 2024-03-13

**Authors:** Cristian Balducci, Martina Roso, Annj Zamuner, Lucia Falcigno, Gabriella D’Auria, Paola Brun, Monica Dettin

**Affiliations:** 1 Department of Industrial Engineering, University of Padova, Via Marzolo 9, 35131 Padova, Italy; cristian.balducci@phd.unipd.it (C.B.); martina.roso@unipd.it (M.R.); annj.zamuner@unipd.it (A.Z.); 2 Department of Civil, Architectural and Environmental Engineering, University of Padova, Via Marzolo 9, 35131 Padova, Italy; 3 Department of Pharmacy, University Federico II of Naples, Via Domenico Montesano 49, 80131 Naples, Italy; falcigno@unina.it (L.F.); gabriella.dauria@unina.it (G.D.); 4 Department of Molecular Medicine, University of Padova, Via A. Gabelli 63, 35121 Padua, Italy; paola.brun.1@unipd.it

**Keywords:** succinyl chitosan, negatively charged chitosan, electrospinning, multilayer matrices, bone tissue engineering, osteoblasts, *S. aureus*, *E. coli*

## Abstract

Chitosan (CS) is a polysaccharide obtainable by the deacetylation of chitin, which is highly available in nature and is consequently low-cost. Chitosan is already used in the biomedical field (e.g., guides for nerve reconstruction) and has been proposed as a biomaterial for tissue regeneration in different body districts, including bone tissue. The interest in chitosan as a biomaterial stems from its ease of functionalization due to the presence of reactive groups, its antibacterial properties, its ease of processing to obtain porous matrices, and its inherent similarity to polysaccharides that constitute the human extracellular matrix, such as hyaluronic acid (HA). Here, chitosan was made to react with succinic anhydride to develop a negatively charged chitosan (SCS) that better mimics HA. FT-IR and NMR analyses confirmed the presence of the carboxylic groups in the modified polymer. Four different electrospun matrices were prepared: CS, SCS, a layer-by-layer matrix (LBL), and a matrix with both CS and SCS simultaneously electrospun (HYB). All the matrices containing SCS showed increased human osteoblast proliferation, mineralization, and gene expression, with the best results obtained with HYB compared to the control (CS). Moreover, the antibacterial potential of CS was preserved in all the SCS-containing matrices, and the pure SCS matrix demonstrated a significant reduction in bacterial proliferation of both *S. aureus* and *E. coli*.

## 1. Introduction

Chitin is the product of the polymerization of N-acetylglucosamine with 1→4 β-glycosidic bonds. Chitin in nature constitutes the material of which the exoskeletons of insects, the claws of crustaceans, fungi, and algae are made. Being the second most widespread polysaccharide on earth after cellulose, it is therefore a material available in large quantities. Chitosan is a homopolysaccharide obtained from the deacetylation of chitin [1,2]. Chitosan is widely available, inexpensive, biocompatible, anti-immunogenic, antibacterial, antifungal, and functionalizable due to the presence of alcoholic and amino groups in its chain [1,3,4]. Chitosan is used in the biomedical field for drug and gene delivery, as a scaffold for the growth of various biological tissues both alone and in combination with other biomaterials, in biosensing, and in the treatment of diseases [5,6,7,8,9].

In this study, we set ourselves the objective of using this economical and available polymer to obtain a mimetic of hyaluronic acid, a component of the extracellular matrix usually included in many scaffolds as well as a component in cosmetics, pharmaceuticals, medicines, and food [10,11,12,13,14,15,16,17]. The most obvious difference between chitosan and hyaluronic acid is in the charges that they present at a physiological pH: chitosan has a polycationic nature while hyaluronic acid has a polyanionic nature.

The effect of the surface charge on osteoblast adhesion, spreading, and migration has long been known [18,19]. The study of Marcolongo M. et al. demonstrated that a negative surface charge promotes osteoblast adhesion by approximately 60% over a neutral surface and that, conversely, a positive surface charge inhibits osteoblast adhesion by about 20% [20].

In the literature, carboxylated chitosan (CMCS) has been obtained by reaction with chloroalkanoic acid or glyoxylic acid. The CMCS, which presented a marked solubility in neutral and basic environments, showed interesting properties for applications in regenerative medicine such as antibacterial properties and the ability to heal wounds as well as lipid-lowering, anti-arteriosclerosis, anti-tumor, anti-coagulant, and hypoglycemic effects [21]. Another type of carboxylated chitosan, succinyl chitosan (SCS), was prepared by Niu X. et al. in DMSO [22]. The synthesis of SCS proposed by Aiping Z. et al. takes place in a 1% wt acetic acid/acetone solution [23]. In the present work, the procedure of Bashir S. has been adapted and consists of carrying out the reaction in acetic acid/methanol and acetone [24].

In previously published studies, the main use of SCS has been focused on obtaining systems/nanoparticles for drug delivery also using the two oppositely charged polymers (CS and SCS) [23,25,26].

In this work, we prepared and studied electrospun matrices composed of alternating layers of CS and SCS (LBL) and compared them with electrospun membranes of CS alone (CS), SCS alone (SCS), or the scaffold produced by the contemporary electrospinning of both polymer solutions (HYB).

The physicochemical analyses confirmed the presence of the carboxylic groups in the obtained SCS. The seeding of human osteoblasts on samples of the different electrospun materials made it possible to identify the LBL and HYB matrices as the best scaffolds capable of promoting cell proliferation, calcium deposition, and gene expression of Vitronectin, Osteopontin, and Runx2.

The antibacterial properties of all the electrospun matrices were confirmed, and the SCS matrix showed a significant load reduction for *S. aureus* and *E. coli* proliferation when compared to the pristine chitosan scaffold.

## 2. Results

### 2.1. Morphology of the Electrospun Matrices

**Figure 1:**
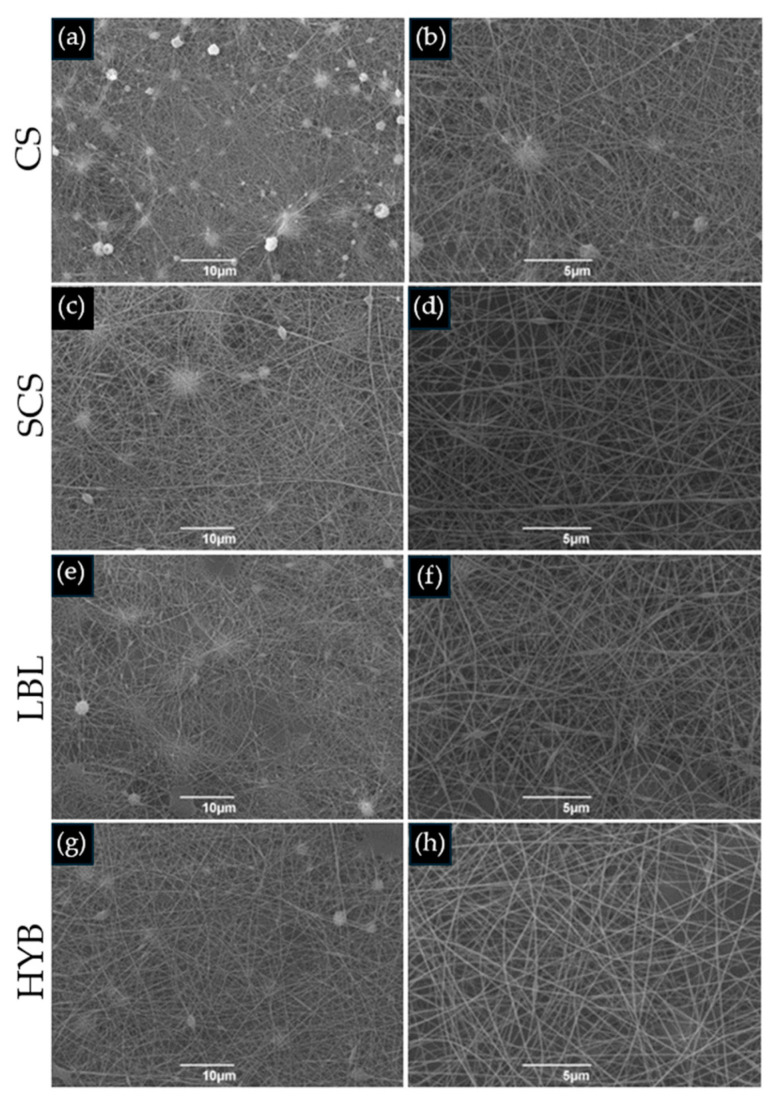
SEM images of electrospun matrices. (**a**,**b**) CS; (**c**,**d**) SCS; (**e**,**f**) LBL; (**g**,**h**) HYB. The images on the left column are at 2000× magnification (scale bar = 10 µm), whilst the right column is at 5000× magnification (scale bar = 5 µm).

The images showing the morphologies of all the electrospun matrices are reported in Figure 1. All the images showed the random fibrous network of the interwoven porous matrices. The pristine CS scaffold showed more defects than the other matrices, maybe due to the absence of the biosurfactant in the electrospun solution. The CS nanofibers showed a lower diameter dimension (0.06 ± 0.01 µm) when compared to the SCS (0.12 ± 0.04 µm), LBL (0.12 ± 0.02 µm), and HYB (0.13 ± 0.03 µm) nanofibers.

### 2.2. Assessment of Chitosan Functionalization

#### 2.2.1. FT-IR Analysis

**Figure 2:**
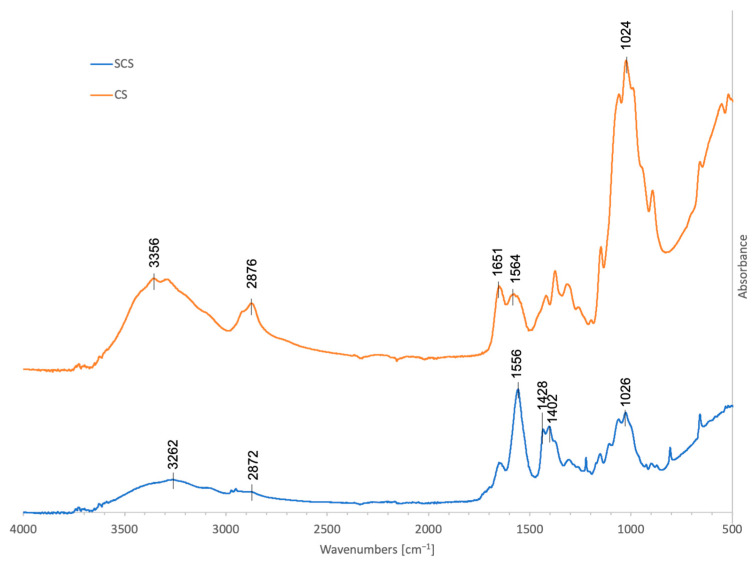
FT-IR spectra in the 450–4000 cm^−1^ region of 70% deacetylated chitosan, 70% deacetylated chitosan functionalized with succinic anhydride (C_4_H_4_O_3_), SCS (blue), and CS (orange).

The results from the FT-IR analysis are reported in Figure 2. The spectrum of pristine chitosan (CS) showed several bands; the 3356 cm^−1^ band is representative of the free O-H bond stretching, overlapping with the N-H bond stretching and H-bonded OH stretching. The band at 2876 cm^−1^ corresponds to the C-H bond asymmetric stretching. The adsorptions at 1651 cm^−1^ and 1564 cm^−1^ are attributed to the stretching vibration of C=O (amide I) and the bending vibration of N-H (amide II) in the acetamide groups (-NH(CO)-CH_3_). The band at 1024 cm^−1^ is related to C-O stretching. Our CS spectra are in line with previously published FT-IR results [27,28].

After the reaction with succinic anhydride (SCS), new contributions appeared in the spectrum: the new bands at 1428 and 1402 cm^−1^ were assigned to the symmetric stretching of the -COO- group, while the band at 1556 cm^−1^ was attributable to the amide II of the succinoyl group (-NH(CO)-CH_2_-CH_2_-COO-) and probably superimposed to the asymmetric stretching of the carboxylate anion [22,29].

According to the SCS FT-IR spectrum, after the reaction, the IR band at 2872 cm^−1^ due to C-H stretching significantly weakened, while the bands at 1556 cm^−1^, 1428 cm^−1^, and 1402 cm^−1^ increased, indicating that the reaction with succinic anhydride occurred.

#### 2.2.2. NMR Analysis

**Figure 3:**
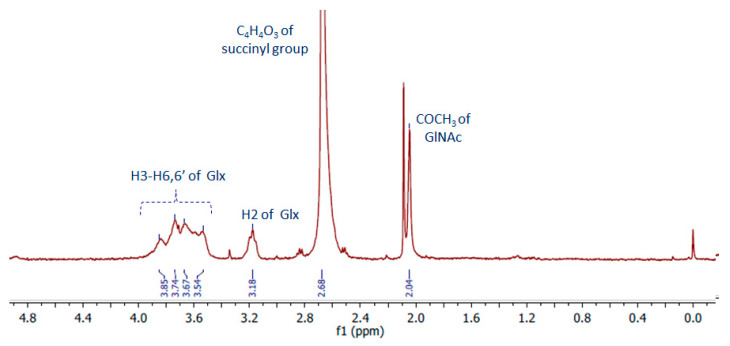
NMR spectrum (400 MHz, T = 343 K) of deacetylated chitosan functionalized with succinic anhydride (C_4_H_4_O_3_), SCS. Glx = Glc or GlNAc.

The 1D spectrum of succinyl-chitosan acquired at 343 K in acidic D_2_O is shown in Figure 3. Moving from left to right, the spectrum shows at the 4.00 ÷ 3.50 ppm region the resonances of H3 ÷ H6,6′ and at 3.18 ppm, H2 protons of chitosan monosaccharides (namely Glc and GlNAc). While the H1 protons of both units are cleared by water pre-saturation, the COCH_3_ group of GlNAc is visible at 2.04 ppm. The functionalization of Glc monosaccharide by the succinyl group is demonstrated by the presence of a large signal at 2.68 ppm, in line with the resonance region expected for NH(CO)CH_2_CH_2_(CO)OH succinyl protons [30].

The acetylation degree of chitosan estimated by the ratio between the integral of the signal at 2.04 ppm (three protons for COCH_3_ of GlNAc) and that of H2-H6,6′ (six protons for each oligosaccharide) is about 0.4 ± 0.1 [31]. This result indicates that the chitosan chains are approximately de-acetylated by ~60 ± 10%, in line with what was found for the free chitosan sample 70 ± 10% (Figure S1). When the ratio of the integral of the succinyl resonances and that of saccharide protons is considered, the value is about 100%, which is higher than expected. This result may be interpreted by considering that the functionalization with the succinyl groups occurs not only on the aminic functions available on the Glc units but also on part of the hydroxyl functions of chitosan. Furthermore, it should be considered that succinyl-chitosan, as well as free chitosan, is a poly-disperse sample in terms of its molecular weight. Therefore, the de-acetylation and succinyl-functionalization that we found refer to values averaged over fractions with molecular weights low enough to be observed by NMR.

### 2.3. Biological Evaluation

#### 2.3.1. Cell Proliferation, Mineralization, and Gene Expression

**Figure 4:**
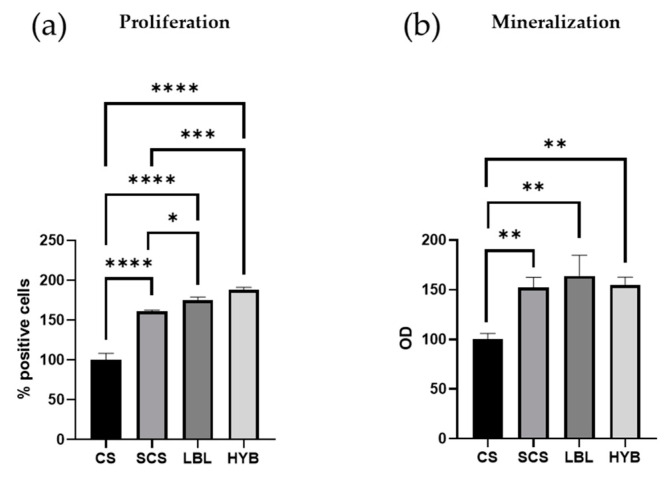
Proliferation and mineralization assays in primary human osteoblast cells cultured for 7 days. Proliferation was determined by loading cells with CFSE fluorescent probe and flow cytometric analysis (**a**). Matrix mineralization was determined using Alizarin staining (**b**). * = *p*-value < 0.05; ** = *p*-value < 0.01; *** = *p*-value < 0.001; **** = *p*-value < 0.0001.

All the electrospun matrices sustained primary human osteoblast proliferation and matrix mineralization. After 7 days in cultures, the osteoblasts showed higher proliferation in all the SCS-containing scaffolds (SCS, LBL, and HYB) compared to the CS scaffold (Figure 4a), with the best results obtained with the HYB matrix, which nearly doubled the pristine CS performance (Figure 4a).

Figure 4b demonstrates a significant enhancement in mineral deposition for the osteoblast cells seeded onto the SCS, LBL, and HYB matrices when compared to the CS control.

**Figure 5:**
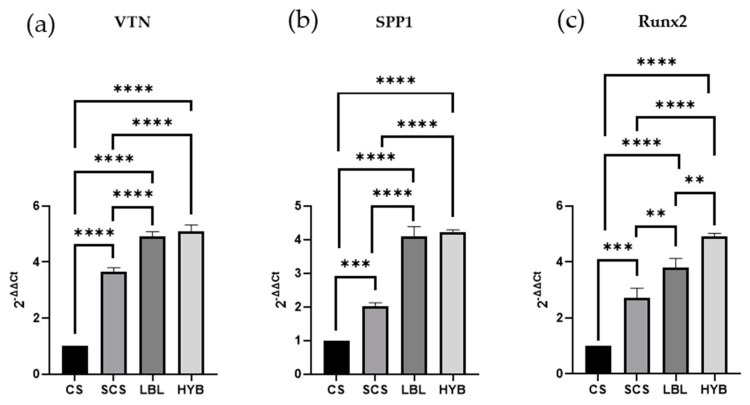
Gene expression assay (qPCR) determined in RNA isolated from osteoblast cells cultured for 24 h on all types of matrices and assessed for the expression of Vitronectin, *VTN* (**a**), Secreted Phosphoprotein 1, *SPP1* (**b**), and Runt-Related Transcription Factor 2, *RUNX2* (**c**). ** = *p*-value < 0.01; *** = *p*-value < 0.001; **** = *p*-value < 0.0001.

To demonstrate the potential ability of the three proposed electrospun scaffolds to induce osteoblast differentiation, we determined mRNA transcript levels specific for three crucial genes (Vitronectin—*VTN*, Secreted Phosphoprotein 1—*SPP1*, and Runt-Related Transcription Factor 2—*RUNX2*). The cells were cultured for 24 h on the different scaffolds. As reported in Figure 5, the SCS, LBL, and HYB matrices showed statistically significant upregulation of all three considered genes. For the gene expression analysis, the best results were observed in the osteoblast cells seeded onto the HYB matrices.

#### 2.3.2. Antibacterial Effects

**Figure 6:**
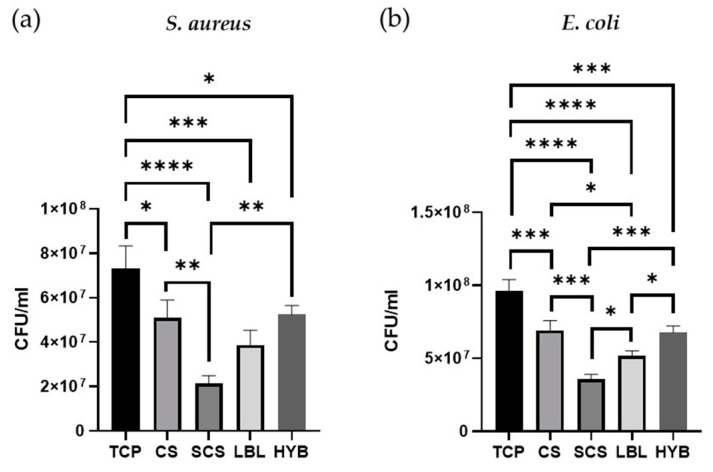
Antibacterial assays, on all types of matrices, with *S. aureus* bacteria (**a**) and *E. coli* (**b**). * = *p*-value < 0.05; ** = *p*-value < 0.01; *** = *p*-value < 0.001; **** = *p*-value < 0.0001.

To ensure that the CS functionalization with succinic anhydride did not affect the intrinsic antimicrobic potential of the pristine CS, two different bacterial species were cultured on all the electrospun matrices, keeping the tissue culture plate (TCP) as a positive control. *S. aureus* (Gram-positive bacteria) and *E. coli* (Gram-negative bacteria) were cultured for 24 h. As shown in Figure 6, all the scaffolds demonstrated better antibacterial properties compared to the TCP (plastic control). Furthermore, the SCS electrospun matrices showed increased antibacterial effects against both *S. aureus* and *E. coli* compared to the pristine CS scaffold.

The LBL matrix also showed greater antibacterial potential against both strains when compared to the CS samples, but the reduction in the bacterial load was significant only in the *E. coli* cultures. The HYB matrix showed the same antibacterial properties observed in the non-functionalized chitosan (CS).

## 3. Discussion

The use of chitosan in biomedical applications is widespread thanks to its bio-compatibility, antimicrobial potential, and the possibility of functionalizing it through its reactive functional groups. In this study, we changed the chitosan polycationic nature into a polyanionic one via a reaction with succinic anhydride. This polyanionic chitosan (SCS) was obtained as a low-cost mimetic of hyaluronic acid, a fundamental component of the extracellular matrix.

The idea of developing layer-by-layer matrices of oppositely charged polysaccharides has already been explored in the literature, especially chitosan/hyaluronic acid electrospun composites [32,33]. Bazmandeh A.Z. et al. proposed a layer-by-layer electrospun matrix of CS-Gelatin/HA as a scaffold for skin regeneration, showing a more favorable cellular adhesion and superior wound healing in vivo. In this scenario, Petrova V.A. et al. demonstrated that the CS-HA electrospun layer-by-layer matrix possessed a slightly better mesenchymal stem cell biocompatibility than CS [34]. Our results confirm that the LBL matrix and the hybrid matrix (HYB) provide better performance regarding osteoblast proliferation, calcium deposition, and gene expression in comparison with the plain matrices (CS or SCS).

Although human osteoblasts possess a negatively charged surface, the conversion from a polycationic to polyanionic nature of chitosan succeeded in promoting cell adhesion, growth, and proliferation, as reported in the study of Marcolongo M. et al., which suggested that a possible difference in the amount of protein adsorbed onto the surface and/or the conformation of that protein layer on the surface could explain the differences in osteoblast adhesion on the negatively charged surfaces.

Furthermore, chitosan’s antimicrobial activity against *S. aureus* (Gram-positive) and *E. coli* (Gram-negative) was not only retained but in the case of the pure SCS matrix also increased, suggesting that the mode of action of chitosan is more complex than simple electrostatic interactions [35,36]. Indeed, since chitosan functionalization affects the total surface charge of the matrices, the observed antibacterial effects likely stem from a dual mechanism: direct bactericidal activity, attributed to alterations in the bacterial membrane charge and cytoplasmic leakage [37], or diminished affinity of the matrices for binding bacterial cells. Our findings show a reduced antibacterial efficacy against Gram-positive bacteria compared to Gram-negative bacteria, suggesting that bacterial wall rigidity and charge density play crucial roles in the interaction with chitosan matrices [38]. On the other hand, the SEM images of the electrospun matrices (Figure 1) revealed minimal variations among the functionalized chitosan matrices regarding the fiber diameters and meshes, thereby ruling out the contribution of structural morphology to their antibacterial properties.

Future studies will certainly focus on the development of a multilayer matrix with more than two layers of chitosan with opposite charges (CS and SCS) to evaluate its biological and antibacterial properties, taking advantage of the possibility of leaving a last layer of SCS, which showed reduced bacterial proliferation with respect to CS. Moreover, to obtain a scaffold with improved performance, the biomechanical properties will be assessed in comparison with the HYB matrix, which will confirm whether electrostatic interactions between the layers will result in increased resistance to matrix flaking.

Considering the work of Filmon R. et al., where the surface of Poly(2-hydroxyethyl methacrylate) was enriched with negative charges through carboxymethylation [39], another interesting cue would be to analyze whether the presence of negative surface charges on SDS-containing matrices induces more significant proliferation and calcium deposition in human primary osteoblasts, as negative charges contribute to the retention of positive calcium ions present in the cell culture medium. In this way, more hydroxyapatite enucleation centers would be formed, making these substrates more supportive for osteoblast adhesion, proliferation, and differentiation with no change in the antimicrobial properties.

## 4. Materials and Methods

### 4.1. Materials

Chitosan 70/1000 (dynamic viscosity: 100 ± 15 mPa∗s, Paragraph S1 of Supplementary Materials) was purchased from Heppe Medical Chitosan GmbH (HMC, Halle, Germany) Generally, chitosan is obtained through the deacetylation of chitin. The chitin for HMC’s chitosan comes mainly from crustacea (*Chionoecetes opilio*). Ethanol, methanol, acetic acid, polyethylene oxide (PEO) Mv ~900,000 (nominal), and succinic anhydride were obtained from Merck KGaA (Darmstadt, Germany). Acetone was provided by VWR International (Radnor, PA, USA).

### 4.2. Methods

#### 4.2.1. Succinyl Chitosan (SCS) Synthesis

SCS was prepared as in [24] with some modifications. Briefly, 0.9 g of chitosan was dissolved in 90 mL of 0.2 M acetic acid solution and stirred for 45 min at 50 °C. After complete dissolution, 45 mL of methanol was added, followed by the drop-by-drop addition of a solution of 2.5 mL of succinic anhydride in 44 mL of acetone. The mixture was left to react for 48 h at 50 °C under magnetic stirring. At the end of the reaction time, the mixture was diluted with 1 M NaOH until it reached a limpid macroscopic appearance and to change the pH from 3 to 7. The limpid solution was left at 50 °C under magnetic stirring for another 24 h. Eventually, ethanol was added to induce SCS precipitation. The SCS was collected by filtration using a gooch n.2. To remove the unreacted reagents, the SCS was extensively washed with ethanol and acetone. Lastly, the SCS was desiccated at 50 °C for 12 h in an under-vacuum stube.

**Figure 7:**
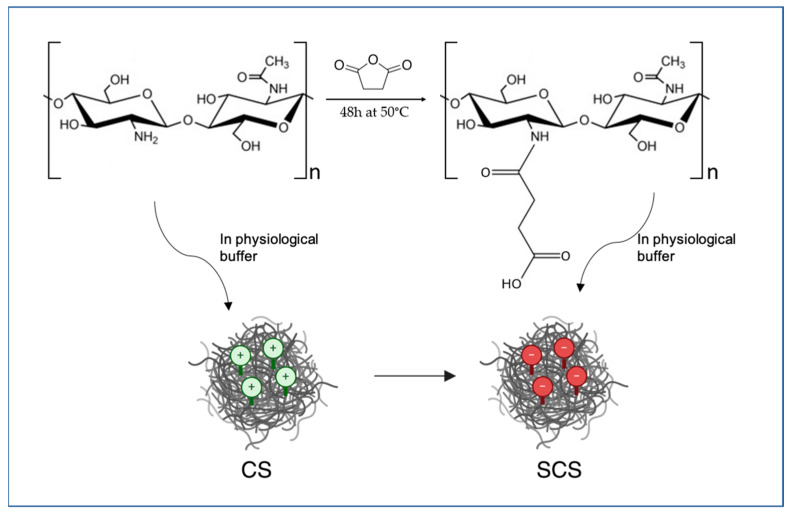
Schematic representation of the chemistry used for SCS preparation.

Figure 7 schematically shows the methodology used to obtain the SCS.

#### 4.2.2. Electrospun Scaffold Preparation

##### CS, SCS, and LBL Matrices

For the CS control matrix, 0.384 g of CS and 0.096 g of PEO (the proportion of CS:PEO was 80:20) were dissolved in 29.52 mL of a 90% acetic acid solution. The total polymer concentration resulted in 1.6% *w*/*w*. The SCS electrospun scaffold was obtained using the proportion of 80:20 between SCS and PEO. Briefly, 0.16 g of SCS and 0.04 g of PEO were dissolved in 5 mL of H_2_O MilliQ, resulting in a final total polymer concentration of 4% *w*/*w*. Eventually, 0.042 g of biosurfactant was added to the final SCS/PEO solution. The LBL scaffold was obtained using both the CS/PEO and SCS/PEO polymer solutions previously described. All the solutions were filtered using a gooch n.2 before being loaded into a 5 mL syringe carrying a 21 G needle. The CS, SCS, and LBL matrices were obtained using a NANON 01-B electrospinning setup (MECC CO., LTD, Fukuoka, Japan). The technical parameters are reported in Table 1. These process parameters resulted from a previous optimization study that allowed the setting of the proper conditions (polymer concentration, flow rate, voltage, temperature, and humidity, as main parameters) to obtain smooth, defect-free fibers with good electrospinnability. This optimization is necessary every time the chemistry of the system polymer/solvent is changed because surface tension, electric conductivity, and viscoelastic forces might be affected by the polymer functionalization, which in turn affects the electrospinnability of the polymer solution.

**Figure 8:**
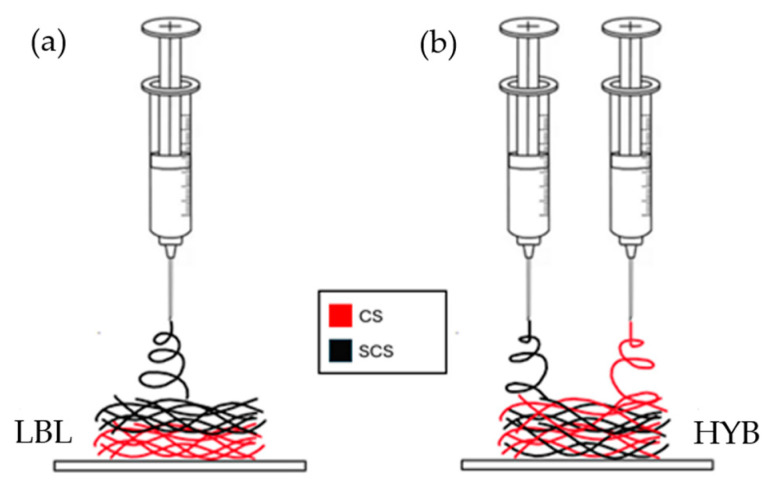
Schematic illustration of the different fiber deposition methods, via electrospinning, for the creation of the LBL matrix (**a**) and the HYB matrix (**b**) on top of aluminum foil.

With respect to the commercial chitosan, the SCS-based solution required a higher polymer concentration, up to 4%wt, to obtain a proper balance between the viscoelastic forces, surface tension, and electrostatic forces. The fibers were collected on aluminum foil and then dried under vacuum for at least 1 h. The LBL scaffold was prepared by electrospinning the CS/PEO solution first (in contact with the aluminum foil), followed by the SCS/PEO solution (on top of the CS/PEO electrospun layer, Figure 8a).

##### Hybrid Matrix

For the hybrid matrix, both the CS/PEO and SCS/PEO solutions were prepared as reported in the previous paragraph. After filtration using a gooch n.2, the CS/PEO and SCS/PEO solutions were loaded into two separate 5 mL syringes carrying a 21 G needle. In this case, both solutions were electrospun simultaneously using two electrospinning apparatuses, as reported in Figure 8. The fibers were collected on aluminum foil covering a rotating stainless steel cylinder collector (500 rpm, rotation speed). The two electrospinning systems were put onto the opposite sides of the rotating collector. All the technical parameters are reported in Table 1.

#### 4.2.3. SEM Analysis

The morphologies of the electrospun matrices were investigated using a Scanning Electron Microscope (SEM, Cambridge Stereoscan 440 SEM, Cambridge, UK). The samples were sputter coated with gold (EMITECH K950× Turbo Evaporator, EBSciences, East Granby, CT, USA). Images were acquired at 2000× and 5000× magnification using an accelerating voltage of 20 kV. The diameter range of the fabricated nanofibers was measured using commercial imaging software (Fiji version 1.0, National Institutes of Health, Bethesda, MD, USA) [40]. Three representative images, in three different areas, were chosen for every sample.

#### 4.2.4. FT-IR Analysis

Fourier-transform infrared analysis was carried out using a Thermo Scientific™ Nicolet™ iS™50 FT-IR Spectrometer (Thermo Fisher Scientific, Waltham, MA, USA), with a diamond crystal as the internal reflection element. The attenuated total reflection (ATR) FT-IR spectra were obtained by plotting the absorbance [%] of the material against the wavenumber [cm^−1^] within the range of 4000–450 cm^−1^ at a resolution of 4 cm^−1^, and the number of scans was set at 32 for each sample.

#### 4.2.5. NMR Analysis

The NMR sample was prepared by dissolving ~ 3.5 mg of succinyl-chitosan in 800 μL of D_2_O (100% D purchased from Sigma-Aldrich) containing 17 μL of HCl 12 M. The 1D proton spectra were acquired at 298 and 343 K using a Bruker Avance NMR spectrometer operating at 400 MHz ^1^H Larmor frequency. The 1D spectra were acquired using 64 scans, an inter-scan delay of 5 s, and water suppression with z-gradients. The TSP at 0.00 ppm was used for spectra internal reference. The acetylation degree and succinyl functionalization of chitosan were obtained by signal integration performed using MESTRENOVA 6.0 software (Mestrelab Research, S.L., Santiago de Compostela, Spain). The acetylation degree was also evaluated by signal integration on free chitosan dissolved in 688 μL of D_2_O (100% D Sigma-Aldrich) containing 12 μL of HCl 12 M at 298 K using a Bruker Avance NMR spectrometer operating at 700 MHz ^1^H Larmor frequency (spectrum reported as Figure S1 of the Supplementary Materials).

#### 4.2.6. Biological Assays

##### Cell Culture

Primary human osteoblast (h-osteoblast) cells were obtained from explants of cortical mandible bone collected during a surgical procedure from a healthy male subject who was 24 years old. The study was approved by the Ethical Committee of the University Hospital of Padova (Aut. 4899/AO/20 of the 5 May 2020). The patient was informed of the study aims and protocol and provided his written informed consent. Bone fragments were cultured at 37 °C in DMEM supplemented with 20% *v*/*v* heat-inactivated fetal bovine serum, 10.000 units mL^−1^ of penicillin, and 10.000 µg mL^−1^ of streptomycin (all purchased from ThermoFisher Scientific, Waltham, MA, USA). The bone fragments were incubated until the cells migrated. At cell confluence, the cells were detached using trypsin-EDTA and cultured in DMEM supplemented with 50 mg mL^−1^ ascorbic acid, 10 nM dexamethasone, and 10 mM β-glycerophosphate (complete culture medium, Merck KGaA, Darmstadt, Germany). The osteoblast phenotype was confirmed using von Kossa staining [41]. In the described experiments, the cells were used between passage 2 and 4 in culture.

##### Cell Proliferation

Primary human osteoblast cells were seeded on the functionalized scaffolds (3 × 10^5^ cells/scaffold) in the complete culture medium. To assess cell proliferation, we first loaded the cells with 25 µM carboxyfluorescein succinimidyl ester (CFSE), a membrane-permeable fluorescent probe that is equally partitioned among daughter cells. Thus, the cells were incubated with CFSE at 37 °C for 10 min in pre-warmed PBS containing 0.1% *v*/*v* bovine serum albumin (Merck KGaA, Darmstadt, Germany). The reaction was stopped by adding 5 volumes of ice-cold culture media. The cells were washed, counted using Trypan blue, and seeded on scaffolds in complete culture medium at 37 °C. Seven days later, the cells were detached and the proliferation was assessed using a BD FACS-Calibur flow cytometer by evaluating the percentage of CFSE-positive cells in 10,000 events.

##### Mineralization Assay

Primary human osteoblast cells were cultured in complete culture medium on functionalized scaffolds for 7 days. The culture medium was renewed every 48 h. At the end of incubation, the cell cultures were washed in PBS and incubated for 30 min at 4 °C with 5% *w*/*v* trichloroacetic acid (Merck). The cell extracts (100 μL) were then combined with HCl 3.6 mM, o-cresolphthalein complexone (o-CPC) 100 μM, and 2-amino-2methyl-1-propanol 0.142 g/mL (all provided by Merck). The absorbance was recorded at 620 nm. The calcium levels were normalized to the protein concentration determined in the cell extracts using the bicinchoninic acid method (Pierce, Thermo Fisher Scientific, Waltham, MA, USA) and reported as the optical density (OD).

##### Gene Expression

Osteoblasts were cultured on different functionalized scaffolds for 24 h at 37 °C in complete culture medium. The cells were then subjected to RNA extraction using the SV Total RNA Isolation System Kit (Promega, Milan, Italy). Contaminated DNA was removed by DNase I digestion. Specific mRNA transcript levels coding human vitronectin (*VTN*), human secreted phosphoprotein 1 (*SPP1*), and human Runt-related transcription factor 2 (*RUNX2*) were quantified using the iTaq Universal SYBR Green One-Step Kit (Bio-Rad, Hercules, CA, USA). The reaction mixture contained 200 nM forward primer, 200 nM reverse primer, iTaq universal SyBR Green reaction mix, iScript reverse transcriptase, and 200 ng total RNA. Real-time PCR was performed using an ABI PRISM 7700 Sequence Detection System (Applied Biosystems, Waltham, MA, USA). Human GAPDH was used as the reference gene. The target and reference genes were amplified with efficiencies near 100%. The oligonucleotides used for PCR are listed in Table 2.

##### Bacterial Cultures

Methicillin-resistant *Staphylococcus aureus* (*S. aureus*, Gram-positive bacterial strain; ATCC 33592) and *Escherichia coli* (*E. coli*, Gram-negative bacterial strain; NCTC 9001) were cultured on Nutrient broth (ThermoFisher Scientific, Waltham, MA, USA). Overnight cultures were diluted to 10^3^ CFU/mL and incubated at 37 °C for 24 h on different scaffolds. At the end of incubation, the cultures were collected, properly diluted, and spread on Nutrient agar plates (ThermoFisher Scientific, Waltham, MA, USA). The plates were incubated at 37 °C for 24 h and the bacterial growth was estimated by counting the bacterial colonies (colony forming units, CFU).

#### 4.2.7. Statistical Analysis

All the data are expressed as the mean ± standard deviation of at least three independent experiments. Statistical analysis was performed using GraphPad Prism software Version 8.4.3 (686) (GraphPad Software Inc., La Jolla, CA, USA), and the statistical significance was calculated using one-way analysis of variance (ANOVA) followed by Tukey’s multiple comparisons test. Statistical significance was considered a *p*-value ≤ 0.05.

## Figures and Tables

**Table 1:** Electrospinning parameters.

Matrix	Solution	Voltage [kV]	Flow Rate [mL/h]	Needle/Collector Distance [cm]	Spin Speed [mm/s]	Temperature [°C]	Humidity [%]	Deposition Time [h]
CS	CS/PEO (80:20) 1.6% *w*/*w*	18	0.4	20	100	22	<20	4
SCS	SCS/PEO (80:20) 4% *w*/*w*	22	0.4	20	200	22	<20	4
LBL	CS/PEO (80:20) 1.6% *w*/*w*	18	0.4	20	100	22	<20	2
	SCS/PEO (80:20) 4% *w*/*w*	22	0.4	20	200	22	<20	2
HYB	CS/PEO (80:20) 1.6% *w*/*w*	11	0.4	20	--	22	<20	2
	SCS/PEO (80:20) 4% *w*/*w*	22	0.4	20	--	22	<20	2

**Table 2:** Oligonucleotides used in qPCR experiments. ^a^ Fw: forward; ^b^ Rv: reverse.

Gene [Accession#]	Sequence
GAPDH	^a^ Fw: 5′-cgggaagcccatcacca-3′
[NM_002046] https://www.ncbi.nlm.nih.gov/nuccore/NM_002046 (accessed on 4 October 2023)	^b^ Rv: 5′-ccggcctcaccccatt-3′
VTN	Fw: 5′-ggaggacatcttcgagcttct-3′
[NM_000638] https://www.ncbi.nlm.nih.gov/nuccore/NM_000638 (accessed on 4 October 2023)	Rv: 5′-gctaatgaactggggctgtc-3′
SPP1	Fw: 5′-aagtttcgcagacctgacatc-3′
[NM_000582] https://www.ncbi.nlm.nih.gov/nuccore/NM_000582 (accessed on 4 October 2023)	Rv: 5′-ggctgtcccaatcagaagg-3′
RUNX2	Fw: 5′-cagtgacaccatgtcagcaa-3′
[NM_001024630] https://www.ncbi.nlm.nih.gov/nuccore/NM_001024630 (accessed on 4 October 2023)	Rv: 5′-gctcacgtcgctcattttg-3′

## Data Availability

The raw data supporting the conclusions of this article will be made available by the authors upon request.

## Supplementary Materials

The supporting information can be downloaded at https://www.mdpi.com/article/10.3390/ijms25063256/s1.
