# Supplementary material for: Multilayer Electrospun Scaffolds of Opposite-Charged Chitosans
Source: Int J Mol Sci. 2024 Mar 13;25(6):3256. doi: 10.3390/ijms25063256 (PMC10969954; doi:10.3390/ijms25063256)
Supplement: Supplementary file 1 [file ijms-25-03256-s001.zip › ijms-2877281-supplementary.pdf]

International Journal of *Molecular Sciences*

MDPI

Supplementary Materials

Multilayer Electrospun Scaffolds of Opposite-Charged Chitosans

Cristian Balducci [1], Martina Roso [1], Annj Zamuner [1,2], Lucia Falcigno [3], Gabriella D'Auria [3], Paola Brun [4] and Monica Dettin [1,*]

1 Department of Industrial Engineering, University of Padova, Via Marzolo 9, 35131 Padova, Italy; cristian.balducci@phd.unipd.it (C.B.); martina.roso@unipd.it (M.R.), annj.zamuner@unipd.it (A.Z.)
2 Department of Civil, Architectural and Environmental Engineering, University of Padova, Via Marzolo 9, 35131 Padova, Italy
3 Department of Pharmacy, University Federico II of Naples, Via Domenico Montesano 49, 80131 Naples, Italy; falcigno@unina.it (L.F.); gabriella.dauria@unina.it (G.D.)
4 Department of Molecular Medicine, University of Padova, Via A. Gabelli 63, 35121 Padua, Italy; paola.brun.1@unipd.it
* Correspondence: monica.dettin@unipd.it; Tel.: +39-049-8275553

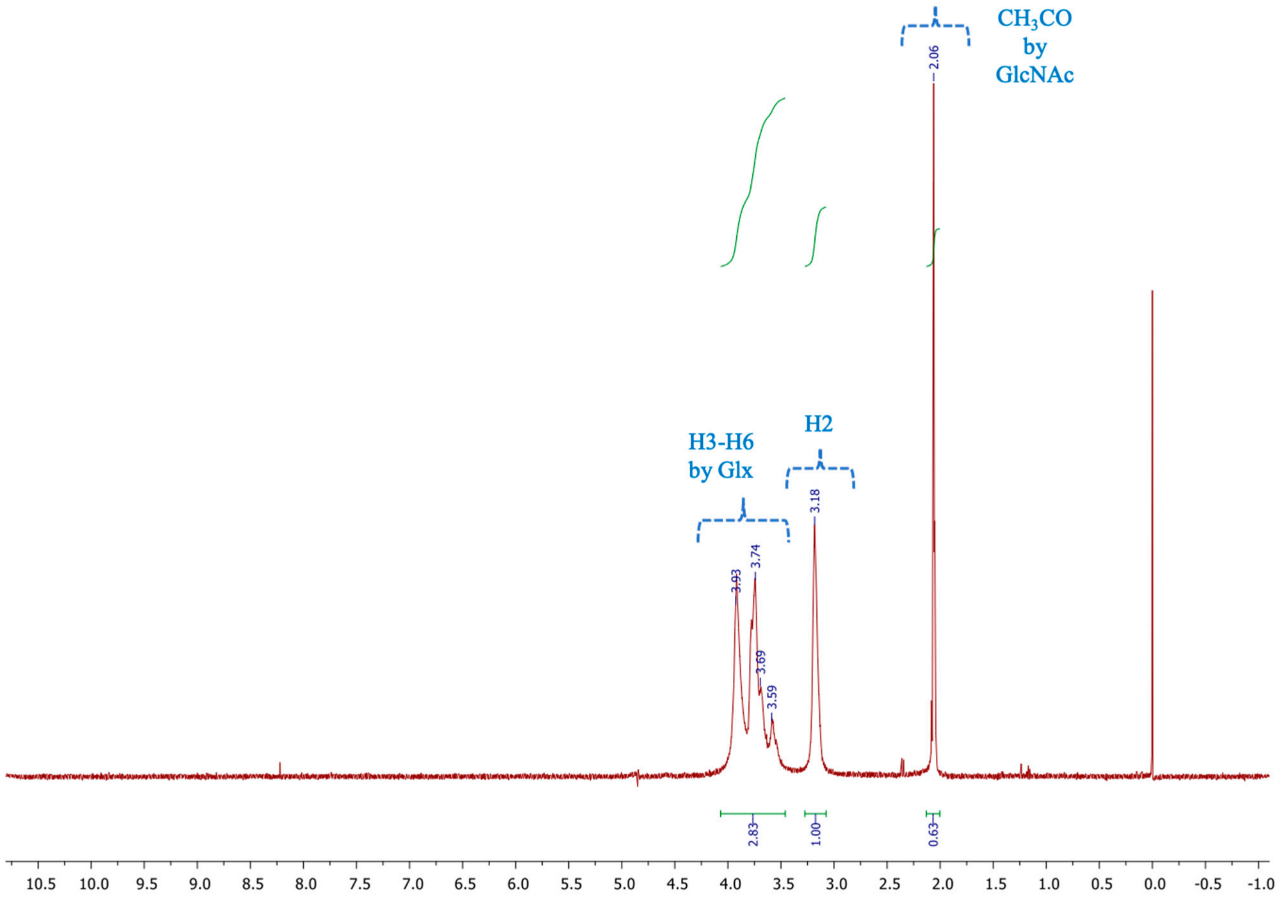

Figure S1. NMR spectrum (700 MHz, T=298K) of free chitosan in acidic D_2O. Glx = Glc or GlNAc.

S1. Materials and Methods

S1.1. Methods

Dynamic viscosity analysis

The dynamic viscosity of a 1% Chitosan aqueous solution in 1% acetic acid was measured by The NμLine® Cone & Plate viscometer (Analytical Technology and Control Ltd, Broadway, Market Lavington, Devizes, Wiltshire SN10 5RQ, United Kingdom) equipped with a cone 2P, in the speed range 10-200 rpm at 20°C.
